# Successful treatment by on-demand glecaprevir and pibrentasvir for hepatitis C flare during R-CHOP in patients with diffuse large B-cell lymphoma: a case report

**DOI:** 10.1186/s12879-021-06091-x

**Published:** 2021-04-27

**Authors:** Machiko Umemura, Goki Suda, Shihori Tsukamoto, Ko Ebata, Shinjiro Takahash, Takashi Sasaki, Sae Nakajima, Koji Hirata, Mariko Ozasa, Masatoshi Takano, Masaki Katagiri, Naoya Sakamoto

**Affiliations:** 1 Sapporo Hokuyu Hospital, Sapporo, Hokkaido Japan; 2 Department of Gastroenterology and Hepatology, Graduate School of Medicine, Hokkaido University, Sapporo, Hokkaido Japan

**Keywords:** Glecaprevir, Hepatitis C virus, Pibrentasvir, Direct-acting antiviral, hepatitis C flare

## Abstract

**Background:**

In patients with hepatitis C virus (HCV) and malignant lymphoma, hepatitis C flare during R-CHOP can result in discontinuation of treatment. However, appropriate therapeutic strategies for managing hepatitis C flare during R-CHOP have not been established, and this issue is complicated by conflicting results regarding the use of direct-acting antivirals in patients with uncontrolled malignancies.

**Case presentation:**

We report the first case of effective and safe treatment with on-demand 8-week glecaprevir and pibrentasvir for hepatitis C flare during R-CHOP in a patient with diffuse large B-cell lymphoma (DLBCL). The patient completed five additional courses of R-CHOP without hepatic toxicity. A complete response of DLBCL and a sustained virological response were observed at 24 weeks after glecaprevir and pibrentasvir completion.

**Conclusion:**

On-demand, direct-acting antivirals could be a novel strategy for managing hepatitis C flare during R-CHOP.

**Supplementary Information:**

The online version contains supplementary material available at 10.1186/s12879-021-06091-x.

## Background

Globally, hepatitis C virus (HCV) remains a major cause of hepatocellular carcinoma and liver-related deaths. HCV infection can cause extrahepatic disorders, including lichen planus, diabetes mellitus, renal dysfunction, and lymphoma [1].

Recently developed direct-acting antivirals (DAAs) have improved the efficacy and safety of anti-HCV therapy compared with interferon-based therapy. Several basic studies, clinical trials, and real-world studies have shown that DAA therapy is highly safe and effective, with minimal drug–drug interactions [2–4]. However, anti-HCV treatment for patients with advanced malignancies remains controversial. DAA treatment is contraindicated in HCV-infected patients with uncontrolled malignancies [5]. In addition, there are no guidelines for the timing of DAA administration with respect to the individual anti-tumor treatment schedule in patients with HCV and cancer. Further, the indications for DAA treatment with these cases remain unknown [5].

HCV infection is associated with lymphoma, especially diffuse large B-cell non-Hodgkin lymphoma and HCV-associated indolent B-cell non-Hodgkin lymphomas [1, 6]. After HCV eradication by anti-HCV therapy, indolent B-cell non-Hodgkin lymphoma regression, especially in marginal zone lymphomas, is sometimes observed, suggesting a link between HCV and lymphoma progression [1, 6, 7]. Compared with non-HCV-positive diffuse large B-cell lymphoma (DLBCL) patients, HCV-positive DLBCL patients were reported to have different characteristics, including elevated LDH and old age [8, 9]. High HCV-RNA viral load is associated with a worse prognosis in patients with diffuse large B-cell lymphoma (DLBCL) [10] and HCV infection causes liver cirrhosis and hepatocellular carcinoma; thus, if possible, proper treatment is required. During standard therapies, including high rituximab, cyclophosphamide, doxorubicin, vincristine, and prednisolone (R-CHOP) for DLBCL, a total of 14–28% of patients experienced grade 3–4 hepatic toxicity [9, 11]. Thus, patients with DLBCL and HCV infection administered with R-CHOP may require careful monitoring for hepatic toxicity, as well as proper management; however, to our best knowledge, no study has evaluated the more recent DAAs; glecaprevir and pibrentasvir for hepatitis C flares during R-CHOP.

Here, we describe successful HCV treatment by on-demand gearlever and pivrentasvir, which is initiated only when hepatic C flare is observed during R-CHOP therapy, in an HCV-infected patient with DLBCL.

## Case presentation

### Case

A 48-year-old man presented to our hospital for cervical lymph node swelling (Supplementary Fig. 1). Cervical lymph node biopsy showed diffuse large cells infiltration in lymphoid follicles. Immunohistochemical staining showed that the large atypical cells were positive for CD20 and CD79a and negative for CD3, bcl-2, bcl-6, and cyclinD1. Ki-67 LI values were graded as more than 90%. In situ hybridization for EBV RNA using the EBER probe showed positive labeling in almost all the atypical cells. Those findings led to the diagnosis of EBV-positive DLBCL, NOS, activated B-cell subtype. Systemic examination, including PET-CT, revealed that the Ann Arbor classification was stage IVA since the mass has spread in the lung and cervical and abdominal lymph nodes.

Table 1 shows the results of blood tests and virological examinations. The International Prognostic Index (IPI), Revised IPI, and the National Comprehensive Cancer Network (NCCN) IPI showed low, good, and low–intermediate risk, respectively. The treatment regimen for the patient was first-line chemotherapy with six courses of R-CHOP.

**Table 1:** Result of blood and urine tests performed before R-CHOP therapy

Parameter	Value	Unit	Reference value	Parameter	Value	Unit	Reference value	Parameter	Value	Unit	Reference value
**Blood cell count**				**Biochemistry**				**Immunology**			
WBC	4720	× 10^3^/μL		TP	9.0	mg/dL	6.5–8.2	CRP	0.79	mg/dL	< 0.30
St	0	%	0.0–19.0	Alb	3.6	mg/dL	3.7–5.5	IgG	2532	mg/dL	820–1740
Seg	89	%	27.0–72.0	α1	3.6	%	1.7–2.9	IgA	457	mg/dL	90–4000
Ly	6	%	18.0–50.0	α2	10.5	%	5.7–9.5	IgM	96	mg/dL	31–200
Mo	5	%	1.0–8.0	β	10.0	%	7.2–11.1	HBsAb	(−)		(−)
Eo	0	%	0.0–7.0	γ	29.5	%	10.2–20.4	HBcAb	(−)		(−)
Ba	0	%	0.0–2.0	BUN	13	mg/ dl	8.0–20.0	HBV-DNA	(−)		(−)
RBC	5	×10^6^/μL	4.38–5.77	Cre	1	mg/ dl	0.65–1.09	HCV Ab	(+)		(−)
Hb	15	g/dL	13.6–18.3	T-bil	0.4	mg/dL	0.3–1.2	HCV-RNA	2.0	LIU/ mL	(−)
Ht		%	40.4–51.9	D-bil	0.1	mg/dL	< 0.4	HCV serotype	Group2		(−)
Plt	295	×10^3^/μL	14.0–37.9	AST	23	U/L	10–40	HIV Ab	(−)		(−)
				ALT	19	U/L	5–45	HTLV-1 Ab	(−)		(−)
**Coagulation**				LDH	211	U/L	120–245				
PT	12	sec	10.0–13.0	ALP	238	U/L	104–338	**Tumor Marker**			
PT%	99	%	80.0–120.0	γ-GTP	104	U/L	< 79	β2MG	2.5	mg/ L	0.9–1.9
PT-INR	1.01	ratio	0.90–1.13	ChE	305	U/L	245–495	sIL-2R	1753	U/ mL	
APTT	28	sec	26.0–38.0	CPK	53	U/L	50–230	SP-D	169.3	ng/ mL	< 110.0
Fib	304	mg/dL	170–410								

*Ab* antibody, *Ag* antigen, *sIL-2R* soluble IL-2 recepter, *β2-MG* β2-microglobulin

The patient was previously diagnosed with HCV infection, with no treatment history for the infection and a history of injecting drug use more than 10 years ago. As shown in Table 1, the HCV genotype was 2, and the HCV-RNA viral load was 2 log IU/mL. Alanine aminotransferase (ALT), aspartate aminotransferase (AST), total bilirubin, PT%, and albumin levels were all within normal ranges and ultrasound and computer tomography imaging did not show liver cirrhosis (Supplementary Fig. 1). In a previous study [12], HCV flare was defined as an increase in HCV-RNA of ≥1 log_10_ IU/mL compared with baseline values and hepatitis flare as an increase in ALT to ≥3 times the upper limit of the normal value.

**Fig. 1:**
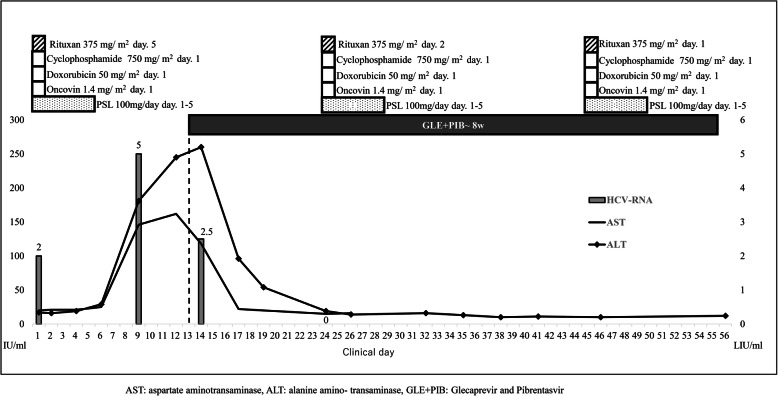
Virologic response and clinical course of glecaprevir and pibrentasvir therapy for HCV and hepatitis flare during R-CHOP for DLBCL. Changes in the serum hepatitis C virus (HCV) titer, alanine aminotransferase (ALT), and aspartate aminotransferase (AST) are shown. HCV, hepatitis C virus; ALT, alanine transaminase

Fig. 1 shows the clinical course for this patient after the initiation of R-CHOP. HCV-RNA levels increased rapidly from 2.0 log_10_ IU/mL at baseline to 5.0 log_10_ IU/mL 9 days after the initiation of the first course of R-CHOP. AST and ALT levels increased rapidly from normal baseline values to 146 U/L and 181 U/L 9 days after R-CHOP initiation and 162 U/L and 245 U/L (CATCAE v5.0, grade 3) at 12 days after R-CHOP initiation, respectively. Based on these findings, the patient was diagnosed with HCV flare and hepatitis flare due to R-CHOP. We initiated 8 weeks of glecaprevir and pibrentasvir for the HCV flare and hepatitis flare 13 days after R-CHOP initiation. As shown in Fig. 1, HCV-RNA decreased rapidly from 5.0 log_10_ IU/mL on day 9 to 2.5 log_10_ IU/mL on day 14. AST and ALT decreased rapidly from 162 U/L and 245 U/L on day 12 to 22 U/L and 96 U/L on day 17, respectively (Fig. 1).

On day 24, when the second course of R-CHOP was initiated, AST and ALT levels normalized and HCV-RNA became undetectable by RT-PCR. The patient completed the 8-week glecaprevir (300 mg/day) and pibrentasvir (120 mg/day) treatment and achieved a sustained virological response at 24 weeks after its completion, without remarkable adverse events.

After the initiation of glecaprevir and pibrentasvir, the patient did not experience hepatic toxicity during the remaining five courses of R-CHOP. Enhanced computed tomography after six courses of R-CHOP revealed a complete response in DLBCL.

## Discussion and conclusions

Recently, several studies have reported the efficacy and safety of concurrent or subsequent anti-HCV therapy in immunochemotherapy (I-CT), including R-CHOP for patients with HCV and malignant lymphoma. Concurrent or upfront concomitant DAAs and I-CT are potential therapeutic approaches [12, 13]; however, a limited number of patients have been treated with I-CT and concurrent DAAs. In addition, anti-HCV treatment for patients with advanced malignancies remains controversial, and DAA treatment is contraindicated in patients with HCV and uncontrolled malignancies [5]. Thus, in general practice, R-CHOP is used without concurrent DAAs for HCV-infected patients with malignant lymphoma.

Administration of DAAs following I-CT—including R-CHOP—is highly safe and effective based on studies involving a relatively large number of patients [12, 13]. However, in the initial I-CT, 60% (23/38) and 18% (7/38) of patients experience any grade of hepatic toxicity and severe hepatic toxicity, respectively [13]. Similarly, Zaky et al. have reported that in HCV-infected patients with lymphoma, R-CHOP causes severe hepatic toxicity at a high rate of 28% (19/68) and that these hepatic toxicities lead to the modification and discontinuation of I-CT, resulting in poor responses to treatment [11]. Thus, the management of hepatic toxicity during R-CHOP in HCV-infected patients with malignant lymphoma is a crucial issue requiring clarification. To the best of our knowledge, the present case is the first evidence for the safety and efficacy of on-demand glecaprevir and pibrentasvir therapy for hepatitis C flare, thereby enabling further R-CHOP without hepatic toxicity. Thus, in addition to concurrent or upfront concomitant DAAs and I-CT, this “on demand” DAAs therapy might be promising approach.

There have been recent reports of HCV reactivation in patients receiving anti-malignancy therapy. In a prospective study, HCV reactivation occurred in 23% (23/100) of patients with HCV infection who were treated with anti-cancer therapy. In addition, of 23 patients with HCV reactivation, 10 patients experienced hepatitis flare, in some cases requiring the discontinuation of the anti-cancer treatment. A multivariate analysis revealed that rituximab and high-dose steroids are significantly associated with HCV reactivation [14]. Thus, R-CHOP, which involves rituximab and high-dose steroids, is considered a high-risk anti-cancer treatment. Moreover, Zaky et al. reported that in patients with DLBCL and HCV infection, R-CHOP is associated with severe hepatic toxicity and shows a high rate of discontinuation. In addition, significantly higher serum HCV-RNA levels after treatment initiation have been observed in patients treated with R-CHOP compared to those treated with CHOP [11].

As described previously, although Deming et al. proposed that DAA treatment is contraindicated in patients with HCV and uncontrolled malignancies, they recommended it in patients with stable cancers or those in remission for at least 3–6 months after cancer treatment [5]. Insufficient cancer therapy might cause poor outcomes; thus, to avoid discontinuation or dose reduction of cancer therapy, we hypothesized that on-demand glecaprevir and pibrentasvir are potential options. Recent favorable treatment outcomes of concurrent DAAs and I-CT for patients with malignant lymphoma and HCV infection [12, 13, 15] support this strategy. Glecaprevir and pibrentasvir are more recent DAAs and approved in many countries (Supplementary Table 1). In Japan, the combined administration of vincristine and glecaprevir and pivrentasvir is not a “precaution for co-administration” and “contraindication for concomitant use” refer to its package insert (https://aconnect.abbvie.co.jp//media/assets/pdf/products/maviret/Maviret_tmpDocument.pdf); however, vincristine is a substrate of P-glycoprotein (P-gp) and concentrations may increase due to inhibition of Pgp by glecaprevir and pivrentasvir and might cause adverse events. Additionally, there is no study regarding combined administration of vincristine and glecaprevir and pivrentasvir; thus, to ensure its safe use, further analysis is warranted. Merli et al. reported nine cases of concurrent administration of DAAs and I-CT. The DAAs regimen of the nine cases consisted of one case of sofobuvir and ribavirin, three cases of sofosbuvir and ledipasvir, and five cases of sofosbuvir and daclatasvir [13]. The safe use of sofosbuvir-based therapies has been reported; thus, these therapies could be used as alternatives for gelcaprevir and pibrentasvir. However, these therapies are adapted to limited HCV genotypes and required longer treatment duration than glecaprevir and pibrentasvir. Thus, further study is warranted.

Several reports have shown that anti-HCV therapy with both IFN and DAAs could improve overall survival or disease-free survival in HCV-infected patients with malignant lymphoma, and HCV infection cause liver cirrhosis and hepatocellular carcinoma [15–17]. Thus, DAA therapy for HCV-infected patients with malignant lymphoma should be considered, with cost and insurance issues varying in each country.

In the present case, after one course of R-CHOP, increases in ALT and HCV-RNA were observed. HCV-RNA increased 1000-fold over baseline levels 9 days after treatment initiation. An immune response to virus-infected hepatocytes is a common cause of viral hepatitis. However, our previous in vitro and in vivo analyses had shown that rapid increases in HCV cause hepatic toxicity [18]. Thus, in this case, in addition to an immune response to virus-infected hepatocytes, as a potential hypothesis, a rapid increase in HCV might cause hepatic toxicity.

After 2 days of glecaprevir and pibrentasvir administration, our patient’s HCV-RNA levels decreased rapidly (a nearly 2.5 decrease); thus, glecaprevir and pibrentasvir showed immediate suppressive effects on HCV replication. This might result in immediate attenuation of hepatic toxicity. Glecaprevir or pibrentasvir monotherapy could decrease HCV-RNA levels by nearly 3 log 24 h after treatment initiation [19]; thus, our patient’s clinical course might be reasonable. Glecaprevir and pibrentasvir have a highly potent anti-HCV ability and induce a shorter treatment duration than previous DAA treatments; this combination might be suitable in R-CHOP-induced HCV flare and subsequent hepatitis.

This case report has a few limitations. Some clinical data, including fibroscan data, were lacking. Additionally, the safety regarding drug-drug interaction between R-CHOP and glecaprevir and pibrentasvir remains unclear. Thus, these should be considered when interpreting the case report results, and a prospective study with a large sample is required to validate the findings.

In conclusion, on-demand glecaprevir and pibrentasvir for hepatitis C flare during R-CHOP in HCV-infected patients with malignant lymphoma might be safe and effective.

## Supplementary Information

**Additional file 1: Supplementary Fig. 1.** Imaging of Cervical lymph nodes and liver. Cervical lymph nodes (a) on CT and (b) on PET-CT. (c) Hepatic image on CT.

**Additional file 2: Supplementary Table 1**. Glecaprevir and piverentasvir approved countries (at March 2021)

## Data Availability

The data that support the findings of this study are available from the corresponding author upon reasonable request.

## Abbreviations

R-CHOP: Rituximab, cyclophosphamide, doxorubicin, vincristine, and prednisolone
ALT: Alanine aminotransferase
AST: Aspartate aminotransferase
HCV: Hepatitis C virus
DAAs: Direct-acting antivirals
DLBCL: Diffuse large B-cell lymphoma
