# Supplementary figures and images for: Successful treatment by on-demand glecaprevir and pibrentasvir for hepatitis C flare during R-CHOP in patients with diffuse large B-cell lymphoma: a case report
Source: BMC Infect Dis. 2021 Apr 27;21:389. doi: 10.1186/s12879-021-06091-x (PMC8077834; doi:10.1186/s12879-021-06091-x)

a.

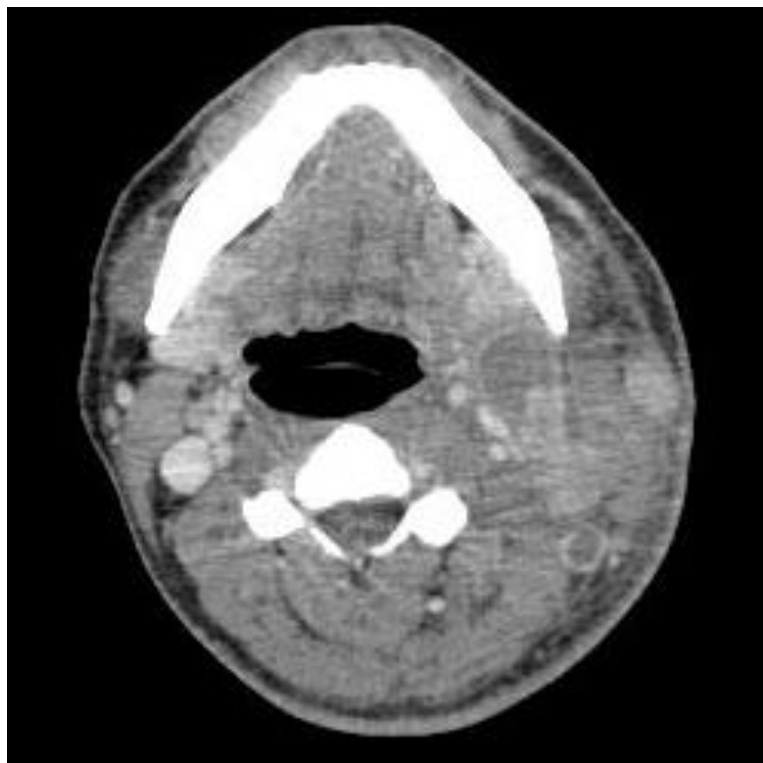

b.

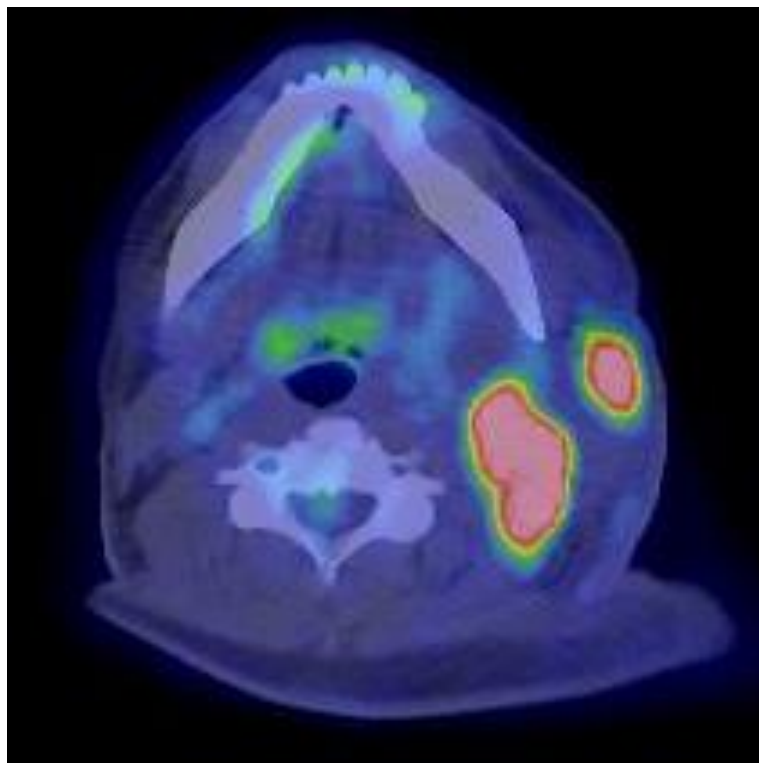

c.

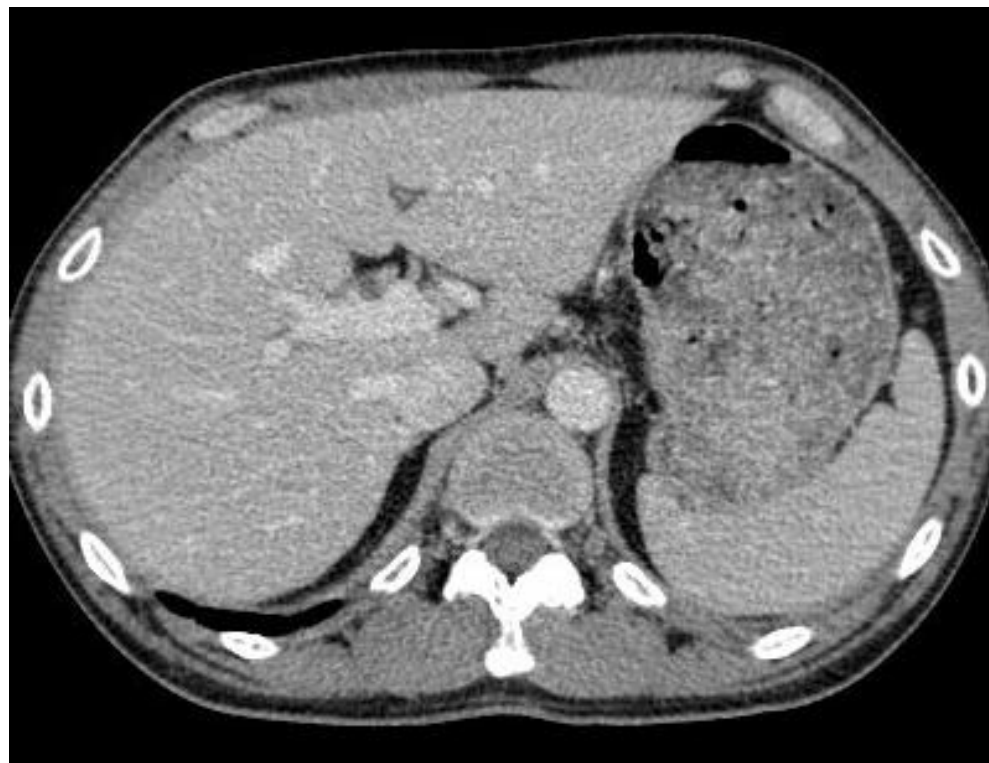

Supplementary Figure1.

Supplement: Supplementary file 1 — Additional file 1: Supplementary Fig. 1. Imaging of Cervical lymph nodes and liver. Cervical lymph nodes (a) on CT and (b) on PET-CT. (c) Hepatic image on CT. [file 12879_2021_6091_MOESM1_ESM.pdf]
