# Supplementary material for: Successful treatment by on-demand glecaprevir and pibrentasvir for hepatitis C flare during R-CHOP in patients with diffuse large B-cell lymphoma: a case report
Source: BMC Infect Dis. 2021 Apr 27;21:389. doi: 10.1186/s12879-021-06091-x (PMC8077834; doi:10.1186/s12879-021-06091-x)
Supplement: Supplementary file 2 — Additional file 2: Supplementary Table 1. Glecaprevir and piverentasvir approved countries (at March 2021) [file 12879_2021_6091_MOESM2_ESM.docx]

**Supplementary Table 1. Glecaprevir and piverentasvir approved**

**countries (at March 2021)**

| **Country** | **Comments** |
| --- | --- |
| US | Approved |
| EU | Approved |
| Albania | Approved |
| Armenia | Approved |
| Argentina | Approved |
| Australia | Approved |
| Belarus | Approved |
| Bosnia and Herzegovina | Approved |
| Brazil | Approved |
| Brunei | Approved |
| Canada | Approved |
| China | Approved |
| Hong Kong | Approved |
| Iceland | Approved |
| Israel | Approved |
| Japan | Approved |
| Kazakhstan | Approved |
| Kuwait | Approved |
| Lebanon | Approved |
| Liechtenstein | Approved |
| Macau | Approved |
| Malaysia | Approved |
| Malta | Approved |
| Mexico | Approved |
| Moldova | Approved |
| Montenegro | Approved |
| New Zealand | Approved |
| Norway | Approved |
| Peru | Approved |
| Puerto Rico | Approved |
| Russia | Approved |
| Saudi Arabia | Approved |
| Serbia | Approved |
| Singapore | Approved |
| South Korea | Approved |
